# Heredity

**Published:** 1889-11-02

**Authors:** E. Symes Thompson

**Affiliations:** Gresham Professor of Medicine. 33, Cavendish-square, W.


### HEREDITY.

In your notice of my lectures on "Heredity" at Gresham College I am reported to have said that "disease follows the sex of the first sufferer, that daughters are apt to inherit a tendency to consumption from their mother, and sons from their father." The statements made in the lecture was that "sons inherited more from the mother than the father, whilst daughters inheriting phthisis from the father passed through a critical time in early womanhood, during which many succumbed, but sometimes outgrew the danger, and, in a considerable number of cases lived on, perhaps with damaged lungs, to old age.

E. Symes Thompson, M.D.,
Gresham Professor of Medicine.
33, Cavendish-square, W.

